# TP53 mutation hits energy metabolism and increases glycolysis in breast cancer

**DOI:** 10.18632/oncotarget.11594

**Published:** 2016-08-25

**Authors:** Hajnalka Harami-Papp, Lőrinc S. Pongor, Gyöngyi Munkácsy, Gergő Horváth, Ádám M. Nagy, Attila Ambrus, Péter Hauser, András Szabó, László Tretter, Balázs Győrffy

**Affiliations:** ^1^ MTA TTK Lendület Cancer Biomarker Research Group, H-1117, Budapest, Hungary; ^2^ Semmelweis University, Department of Medical Biochemistry, H-1094, Budapest, Hungary; ^3^ Semmelweis University, Second Department of Pediatrics, H-1094, Budapest, Hungary

**Keywords:** glycolytic efficiency, breast cancer, Warburg effect, next generation sequencing

## Abstract

Promising new hallmarks of cancer is alteration of energy metabolism that involves molecular mechanisms shifting cancer cells to aerobe glycolysis. Our goal was to evaluate the correlation between mutation in the commonly mutated tumor suppressor gene *TP53* and metabolism. We established a database comprising mutation and RNA-seq expression data of the TCGA repository and performed receiver operating characteristics (ROC) analysis to compare expression of each gene between *TP53* mutated and wild type samples. All together 762 breast cancer samples were evaluated of which 215 had *TP53* mutation. Top up-regulated metabolic genes include glycolytic enzymes (*e.g. HK3, GPI, GAPDH, PGK1*, *ENO1*), glycolysis regulator (*PDK1*) and pentose phosphate pathway enzymes (*PGD, TKT*, *RPIA*). Gluconeogenesis enzymes (*G6PC3, FBP1*) were down-regulated. Oxygen consumption and extracellular acidification rates were measured in *TP53* wild type and mutant breast cell lines with a microfluorimetric analyzer. Applying metabolic inhibitors in the presence and absence of D-glucose and L-glutamine in cell culture experiments resulted in higher glycolytic and mitochondrial activity in *TP53* mutant breast cancer cell lines. In summary, *TP53* mutation influences energy metabolism at multiple levels. Our results provide evidence for the synergistic activation of multiple hallmarks linking to these the mutation status of a key driver gene.

## INTRODUCTION

Chronic cell proliferation requires corresponding adjustment of energy metabolism in order to fuel rapid cell growth and division. Normal cells process glucose to pyruvate via glycolysis, and thereafter to CO_2_ in the mitochondria via an affiliated ATP producer mechanism, the oxidative phosphorylation. A feature of cancer cell metabolism first observed by Otto Warburg in 1956 involves their capability to reprogram glucose metabolism (increase glycolysis rate) in aerobic conditions [1]. By limiting their energy metabolism this leads to a state that has been termed “aerobic glycolysis” (also known as the “Warburg effect”). Increased rate of glycolysis allows the diversion of glycolytic intermediates into various biosynthetic pathways that are required for assembling new cells [2]. The very same process can also be observed in normal, rapidly dividing embryonic cells [3]. In addition, high glycolysis in tumor cells correlates with the degree of tumor malignancy, its ability to form metastasis and has been showed to be related to resistance against chemo- and radiotherapy treatments [4, 5].

Driver genes behind this process include *GLUT1*, which enables the uptake of glucose necessary for the process and the *HIF1* alpha and *HIF2* alpha transcription factors responsible for the initiation of glycolysis by triggering the expression of multiple target genes [6]. Altered energy metabolism is in close connection to the six established hallmarks of cancer (activation of oncogenes, loss of tumor suppressors, replicative immortality, evasion of apoptosis, induction of neoangiogenesis and of metastases). Moreover, transformed energy metabolism is proving to be so widespread in cancer cells that it has been recently accepted as a new hallmark of cancer [7].

*TP53* is frequently mutated in human cancers; somatic *TP53* mutations occur in almost every type of human tumors and in over 50% of all tumors [8]. As the “guardian of the genome”, TP53 plays a key role in maintaining genomic stability and tumor prevention [9]. In breast cancer, genes correlated to *TP53* mutation predict the risk of tumor relapse [10]. *TP53* positively correlates to *MKI67* expression and thus to tumor proliferation [11]. In other tumors with low *TP53* mutation rates, TP53 is often inactivated by alternative mechanisms [12]. TP53 can also impact glycolysis through mechanisms including transcriptionally repressing the expression of glucose transporters, down-regulating rate limiting enzymes of glycolysis, decreasing the expression of transporters responsible for lactate extrusion and negatively regulating the AKT/mTOR and NF-κB signaling pathways [13]. TP53 can also modulate expression of glycolytic enzymes like phosphoglycerate mutase [14] and TIGAR, a stimulator of gluconeogenesis and the pentose phosphate pathway [15]. Furthermore it was found that TP53 has effect on the ATP level by influencing the mitochondrial energy production via disruption of the SCO2 gene [16].

In our research our conceptual idea was that we assume that the mutations in *TP53* will not only influence genes because its tumor suppressor properties but will also directly impact enzymes of glycolysis and citrate cycle involved in the energy metabolism of the cells. This property would enable to simultaneously influence multiple key hallmarks of cancer by a single genetic mutation. To validate this hypothesis, we utilized a large breast cancer cohort where both mutation state and gene expression levels were available simultaneously for each gene. To evaluate the expression of genes involved in metabolic cycles we designated sub-cohorts based on *TP53* mutation state (using coding and silent mutations as well). Using these datasets we demonstrate that genes driven by *TP53* mutation status have a high relevance in establishing the Warburg effect. In our *in vitro* experiments we analyzed metabolic profiles in three breast cancer cell lines with different *TP53* status (wild type MCF7 and mutated *TP53* in MDA-MB-231 and JIMT-1) and demonstrate that glycolytic activity is concomitant with *TP53* status.

## RESULTS

### Assessment of energy metabolism in wild type and mutated clinical samples

ROC analysis was performed to identify genes associated with *TP53* mutation. We identified 2,178 genes positively expressed and 1,584 genes negatively regulated genes in the mutant *TP53* cohort compared to the wild type *TP53* cohort at p<0.05. When evaluating the type of mutation, out of 215 patients with a mutation in TP53, 210 samples had a coding mutation and only 5 samples had a non-coding mutation. Therefore, when running the analysis using the coding mutations and all mutations, the results were almost identical. We did not performed the analysis using the silent mutations only due to the very low sample number.

The entire gene list was analyzed based on carbon metabolism pathways form the Kyoto Encyclopedia of Genes and Genomes (KEGG database) [17]. The up-regulated gene group had connection with two pathways: glucose degradation (glycolysis) and pentose phosphate pathway, plus contained a glycolysis regulator. The down-regulated genes were connected to two pathways, gluconeogenesis and fatty acid oxidation pathway (FAOP), and a mitochondrial regulator gene was also included.

### Expression changes of the most important genes

**Figure 1:**
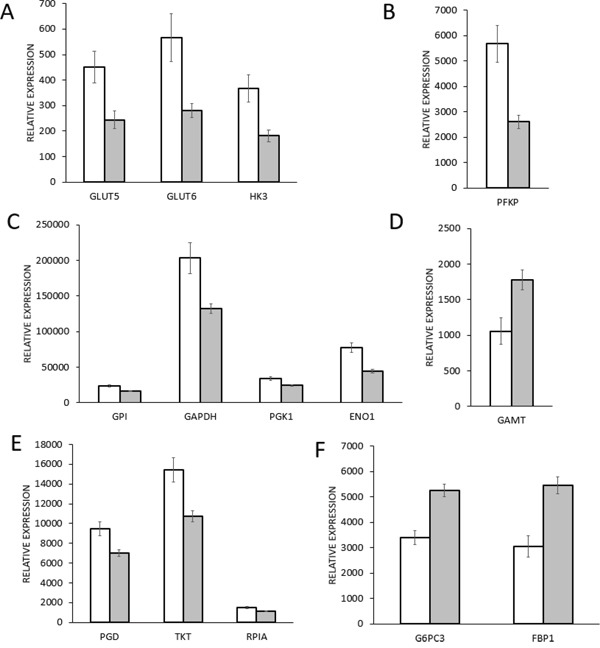
Gene expression of glycolysis enzymes in mutant *TP53* cohort (mutTP53, n=215) and in wild type *TP53* cohort (wtTP53, n=547) breast cancer samples White columns show the mutTP53 patient group gene expression level and grey columns show the wtTP53 patient group. Data are presented as mean ± 95% confidence interval. **A.** *SLC2A5*: Solute carrier family 2 (*GLUT5*); *SLC2A6*: Solute carrier family 2 (*GLUT6*); *HK3*: Hexokinase 3, **B.** *PFKP*: Phosphofructokinase P, **C.** *GPI*: Glucose-6-phosphate isomerase; *GAPDH*: Glyceraldehyde-3-phosphate dehydrogenase; *PGK1*: Phosphoglycerate kinase 1; *ENO1*: Enolase 1, **D.** *GAMT*: guanidinoacetate N-methyltransferase, **E.** *PGD*: 6-phosphogluconate dehydrogenase; *RPIA*: Ribose 5-phosphate isomerase; *TKT*: Transketolase, **F.** *G6PC3*: Glucose-6-phosphatase catalytic 3, *FBP1*: Fructose-1,6-biphosphatase 1.

ROC results for the 16 top ranking genes that had connection to cellular energy metabolisms are listed in Table 1. The glycolysis pathway included eight up-regulated enzymes, two glucose transporters and six carbohydrate modulator enzymes. The glucose transporters *GLUT5* and *GLUT6* mRNA expression were almost twofold higher in the mutTP53 breast cancer patients than in wtTP53 patients (Figure 1A). Hexokinases (HK) perform the first step in most glucose metabolism pathways. mRNA expression of one izozyme of HK (*HK3*) was twofold higher in the mutTP53 patient group (Figure 1A). Phosphofructokinase (PFKP) enzymes process the third step of glycolysis, and its isoform gene expression was twofold higher in the mutTP53 group (Figure 1B). Glucose-6-phosphate isomerase (*GPI*) gene product performs the second step of glycolysis, and its expression was one and a half times as much in the mutTP53 patient group than in the wtTP53 patient group. The protein product of *GAPDH* gene converts D-glyceraldehyde 3-phosphate into 3-phospho-D-glyceroyl phosphate via the fifth step of glycolysis, and its expression was one and a half times higher in the mutTP53 group. The phosphoglycerate kinase 1 (PGK1) converts 1,3-diphosphoglycerate to 3-phosphoglycerate and phosphorylates ADP to ATP (substrate level phosphorylation) during the sixth step, its expression was almost one and a half times higher. Enolase 1 (*ENO1*) catalyze the ninth step of glycolysis, its expression was one and a half higher in the mutTP53 group (Figure 1C). Pyruvate dehydrogenase kinase (*PDK*1) phosphorylates and inactivates pyruvate dehydrogenase complex (PDHc) thus decreases glycolytic flux to tricarboxylic acid cycle [18], its expression was more than one and a half times higher in the mutTP53 group. Pentose phosphate pathway (PPP) is a glycolysis parallel metabolic pathway that generates materials for the reductive biosynthesis reactions. Three up-regulated genes participate in PPP including 6-phosphogluconate dehydrogenase (*PGD*), ribose 5-phosphate isomerase (*RPIA*), and transketolase (*TKT*) – each of these genes was up-regulated (Figure 1E).

**Table 1:** Energy metabolism genes with the highest AUC value identified in mutant TP53 breast cancer patients

GENE	CODED PROTEIN	AUC	P-VALUE
**GLYCOLYSIS ENZYMES**			
*SLC2A5*	Solute carrier family 2, member 5 (*GLUT5*)	0.70	1.9e-17
*SLC2A6*	Solute carrier family 2, member6 (*GLUT6*)	0.73	4.1e-21
*HK3*	Hexokinase 3	0.73	6.8e-21
*GPI*	Glucose-6-phosphate isomerase	0.70	2.6e-16
*PFKP*	Phosphofructokinase P	0.70	1.6e-17
*GAPDH*	Glyceraldehyde-3-phosphate dehydrogenase	0.68	5.7e-14
*PGK1*	Phosphoglycerate kinase 1	0.72	1.1e-19
*ENO1*	Enolase 1	0.77	1.1e-27
**GLUCONEOGENESIS ENZYMES**			
*G6PC3*	Glucose-6-phosphatase catalytic 3	−0.70	−5.4e-17
*FBP1*	Fructose-1,6-biphosphatase 1	−0.70	−4.2e-17
**REGULATORS OF ENERGY METABOLISMS GLYCOLYSIS REGULATORS**			
*PDK1*	Pyruvate dehydrogenase kinase 1	0.70	4.3e-17
**MITOCHONDRIAL REGULATORS**			
*GLS2*	Mitochondrial glutaminase 2	−0.73	−7.0e-22
**FATTY ACID OXIDATION PATHWAY REGULATORS**			
*GAMT*	Guanidinoacetate N-methyltransferase	−0.71	−8.7e-19
**PENTOSE PHOSPHATE PATHWAY ENZYMES**			
*PGD*	6-phosphogluconate dehydrogenase	0.67	4.4e-13
*RPIA*	Ribose 5-phosphate isomerase A	0.69	2.9e-15
*TKT*	Transketolase	0.69	8.1e-16

Positive AUC values correspond to up regulation and negative AUC values to down regulation

Genes enclosed in the down-regulated groups included two out of three key enzymes of gluconeogenesis, glucose-6-phosphatase (G6P) and fructose-1,6-bisphosphatase (FBP1). Gluconeogenesis is a parallel pathway compared to glycolysis, its enzymes perform the reversible conversion of carbohydrate substrates to glucose [18]. The *G6PC3* gene encodes the catalytic subunit of G6P. RNA expression of both of the two gluconeogenesis enzymes was almost half as much in the mutTP53 group as in the wtTP53 patient group (Figure 1F). The analysis showed other down-regulated genes, such as the *GLS2* and *GAMT* (Figure 1D). *GLS2* gene encodes a mitochondrial glutaminase that is an enzyme in glutaminolysis, and promotes mitochondrial respiration and increases ATP generation [19]. RNA expression of *GLS2* was less than half in the mutTP53 group than in the wtTP53 patient group. The GAMT gene encoded guanidinoacetate N-methyltransferase that is part of the creatine synthesis, and may have a TP53-dependent regulator function of fatty acid oxidation [20].

**Figure 2:**
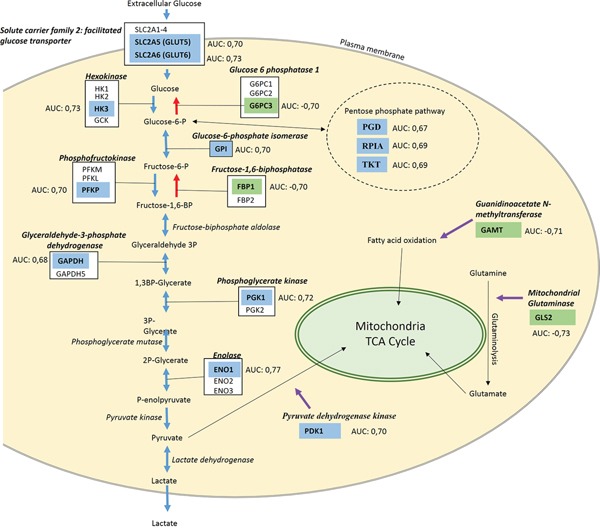
Summary of genes involved in the glycolytic processes affected by *TP53* mutation Blue boxes indicate genes up regulated in *TP53* mutant samples, green boxes indicate down regulated genes. Blue arrows mark the glycolysis pathway, red arrows indicate the gluconeogenesis pathway and purple arrows indicate regulators of related metabolism pathways.

In summary, mutant TP53 influenced genes in the glycolysis and other energy metabolic pathways at multiple levels. The expression changes for each of the involved genes are summarized in Figure 2.

### Cell culture setup

To investigate the effect of *TP53* mutation, we compared the metabolic activity between wtTP53 and mutTP53 breast tumor cell lines. First, *TP53* mutations were validated by sequencing the appropriate exon. Location of the mutations were from the *Catalogue of somatic mutations in cancer* (COSMIC) database. In case of JIMT-1 the amino acid exchange from arginine (R, CGG) to tryptophan (W, TGG) is located in the seventh exon at amino acid #248 (Supplementary Figure 1B). In case of MDA-MB-231 the amino acid exchange from arginine (R, AGA) to lysine (K, AAA) is located in the eighth exon at amino acid #280. The *TP53* mutation in MDA-MB-231 cell line can explain the previously described resistance of the cell line against antimetabolites [21]. We have determined all 11 exons in MCF7 to validate the wild type sequence (data not shown).

### Gene expression in the cell lines

The expression difference for a selected set of ten genes were further validated by RT-PCR in the cell lines. For nine out of ten genes (ENO1, FBP1, HK3, G6PC3, GAPDH, GLS2, PGD, PGK1, and TKT) the expression change was parallel in the clinical samples and in the cell lines (Supplemental Figure 2.).

### Oxygen consumption in DMEM based medium

**Figure 3:**
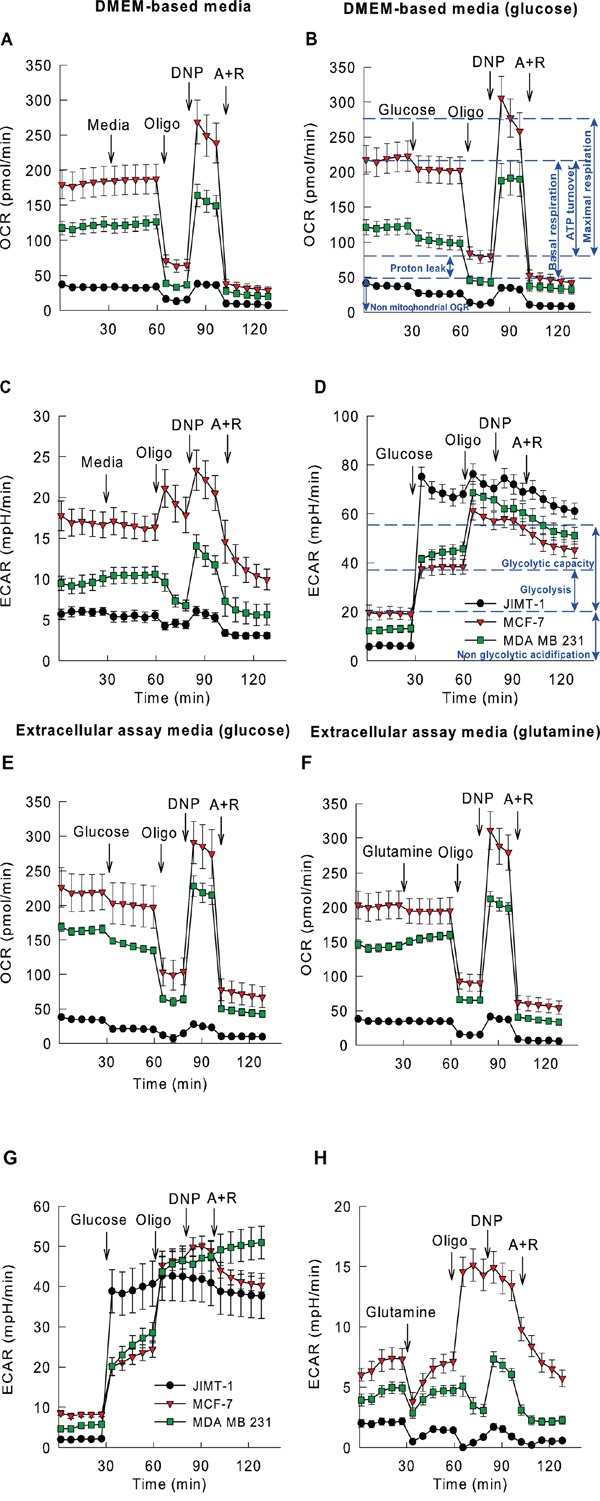
Higher glycolytic and mitochondrial activity observed in *TP53* mutant cell lines Oxygen consumption (OCR) and extracellular acidification rates (ECAR) of JIMT-1, MCF-7 and MDA-MB-231 breast cancer cells (legends are displayed in panels F and I) measured in DMEM-based media **A-D.** or Extracellular assay media. **E-H.** OCR and ECAR were determined using a microfluorimetric Seahorse XF96 Analyzer. Cells were measured i) in the absence (A,C) or presence (B,D) of D-glucose in DMEM-based media and ii) in the presence of D-glucose (E,G). or L-glutamine (F,H). as single substrates in Extracellular assay media. Bioenergetic and glycolytic functions were assessed upon addition of various metabolic inhibitors (e.g. oligomycin (“oligo”), an inhibitor of the mitochondrial FoF1 ATP-synthase or antimycin A + rotenone (“A+R”), inhibitors of the mitochondrial respiratory chain). The highest OCR was exhibited with the uncoupler 2,4-dinitrophenol (DNP) given. D-glucose (10 mM), L-glutamine (2.5 mM), oligomycin (2 μM), 2,4-dinitrophenol (100 μM), antimycin A + rotenone (1-1 μM; all in final concentrations) were given as displayed on plots. Results are averages of measurements performed on three consecutive preparations of each individual cell culture (N=12-24, ∼20,000 cells/well); individual data points are represented as mean ± S.E.M. The blue dashed lines in B and D serve as an interpretation guideline – they represent a schematic average value of multiple time point measurements and the double arrow indicate the key oxygen consumption (B) and extracellular acidification (D).

There were significant differences in absolute oxygen consumption among the investigated cell lines. In terms of oxygen consumption, MCF-7 cells exhibited the highest basal rate followed by MDA-MB-231 and JIMT-1 cells, in decreasing order. All the cell lines responded to oligomycin with a decrease of oxygen consumption rate (OCR) showing that oxidative phosphorylation had been active in all the cases (Figure 3A). If data were expressed as percentages of the basal oxygen consumption rate, then, in the presence of oligomycin, even in JIMT-1 cells, there was a 70% decrease of the oxidation rate (data not shown). Reserve respiratory capacity followed the same pattern as the baseline respiration; far the highest value was found for MCF-7 and the lowest for JIMT-1 (Figure 3A). Mitochondrial respiration was found responsible for at least 80% of the total oxygen consumption.

Administration of glucose to these cancer cell lines resulted in significant decrease in oxygen consumption in each case; the most pronounced effect was exhibited by JIMT-1 cells (32.9±3.9 %) (Figure 3B). Addition of an uncoupler significantly augmented the rate of oxidation in both MDA-MB-231 and MCF-7 cells (without glucose) (Figure 3A). However, in JIMT-1 cells uncoupler dinitrophenol (DNP) stimulated respiration showed a higher rate relative to the one in the presence of glucose, although it was still beneath the baseline (Figure 3B).

### Extracellular acidification in DMEM based medium

The basal rates of pH change showed remarkable parallelisms to basal rates of oxygen consumptions. The highest rate of acidification was measured in MCF-7 cells whereas the lowest rate was found in JIMT-1 cells (Figure 3C). In DMEM based medium the energy donor substrates are mainly those amino acids that are capable of being decomposed to TCA cycle intermediates. Consequently, acidification in this medium can principally be attributed to the CO_2_ that is released during (oxidative) decarboxylation. Under these conditions oligomycin slows acidification down as it hampers mitochondrial oxidation involving the TCA cycle and reactions of oxidative decarboxylation.

When DNP alleviated the oligomycin mediated inhibition, TCA cycle could start working with maximal speed and pH changes could turn to be of the highest rates. Inhibition of mitochondrial metabolism drove the rate of acidification well below the baseline (Figure 3C). Glucose significantly enhanced the extracellular acidification rate (ECAR) in the DMEM based medium for all the cell lines (Figure 3D). The most pronounced elevation was observed in JIMT-1 cells where the rate of acidification was increased by a factor of twelve; in MCF-7 cells glucose stimulated acidification resulted only in a less than twofold increase. In the presence of glucose, MDA-MB-231 and MCF-7 cells responded to oligomycin with a significant further acidification, while in JIMT-1 cells the effect of oligomycin was not significant (Figure 3D).

### Oxygen consumption in extracellular assay medium

Cells were kept in energy substrate-free (EC) medium for 2.5 hours before measurement. Interestingly this starvation period did not affect significantly the basal rate of respiration indicating that cells could fulfill their energy requirements by mobilizing their own energy stores; probably autophagic mechanisms have also been activated. The basal rate of O_2_ consumption was thus the highest in MCF-7 cells, followed by MDA-MB-231 and JIMT-1 cells (Figure 3E) (similarly to that of experiments performed in DMEM-based media). All of the cell lines responded well to oligomycin; the magnitude of the decline of respiration was found to be approximately 60% indicating that mitochondria were well coupled (Figure 3E). Uncoupler mediated stimulation of respiration was of the smallest magnitude in JIMT-1 cells, even when oxygen consumption was normalized to the baseline.

Glucose triggered a significant drop in O_2_ consumption in all of the cell lines; the greatest effect was observed in JIMT-1 cells (48.5±5.5 %) (Figure 3E). All of the cell lines responded to oligomycin in a similar fashion. The rate of reserve respiration measured in the presence of an uncoupler was the smallest in JIMT-1 cells; in the presence of glucose it has not even reached the basal respiratory rate.

Glutamine induced a small (<20%), but significant increase in O_2_ consumption in JIMT-1 and MDA-MB-231 cells (Figure 3F). The uncoupler DNP enhanced the rate of respiration to higher than the baseline in all of the cell lines. Although, JIMT-1 cells exhibited the smallest elevation of the rate, but even their oxygen consumption was now more intense than the one represented by the baseline indicating that glutamine was indeed a good respiratory substrate in the absence of glucose for this mainly glycolytic cell line (Figure 3F).

### Extracellular acidification in extracellular assay medium

In EC medium the smallest rate of acidification was measured for JIMT-1 cells. There was no significant difference between the acidification rates of MDA-MB-231 and MCF-7 cells. However, glucose augmented the acidification rate of JIMT-1 cells 15-fold (Figure 3G) indicating their extreme dependence on glycolysis. Similarly to data collected in DMEM based medium, to a lesser extent glycolysis also accelerated in MDA-MB-231 and MCF-7 cell lines upon addition of glucose and this acidification was further stimulated by oligomycin. The maximal acidification capacity was similar in the three cell lines investigated. Glutamine enhanced the rate of oxidation, but this elevation was not accompanied by an elevation of the rate of acidification (Figure 3H). This lack of acidification can potentially be explained by the release of ammonia due to glutaminase and glutamate dehydrogenase reactions.

## DISCUSSION

Cancer cell lines show many characteristic tumor-specific alterations in glycolysis. Cancer cells may consume more than 10 times more glucose than their normal counterparts [22]. High rate of glucose uptake can be detected and used as a diagnosis of cancer. Accelerated glucose metabolism in cancer is associated with increased expression of glucose transporters, especially GLUT1. TP53 can repress at the transcriptional level GLUT1-4 gene expression that results in decreased glucose uptake [23]. In our study, multiple genes in metabolic pathways were significantly linked to *TP53* mutation including Hexokinase 3, Phosphofructokinase, Platelet (PFKP) and Pyruvate dehydrogenase kinase. Under stress condition, TP53 can gear up glucose uptake by increasing hexokinase transcription [24]. Glucose molecules have to be phosphorylated intracellularly by hexokinases (HK). HK II is upregulated in many cancer cell types [25]. Phosphofructokinase 1 (PFK1) is the rate limiting step of glycolysis. In tumor cells a post-translationally modified form is expressed frequently [26]. One the major mechanisms responsible for the Warburg phenomenon is that HIF-1α and TP53 have effect on the regulation of pyruvate dehydrogenase complex [27].

TP53 can promote fatty acid oxidation when available glucose levels are low to generate ATP in the tricarboxylic acid (TCA) cycle using alternative energy sources. The TP53-dependent regulation of fatty acid oxidation pathway occurs by activation of GAMT [28]. Gluconeogenesis, a reverse glycolytic pathway, generates glucose from small noncarbohydrate precursors. Zhang et al. [29] showed roles for TP53 in the down-regulation of gluconeogenesis both *in vitro* in colon and liver cancer cells and *in vivo*, by down regulation the gene expression of *PCK* and *G6PC*, the rate limiting enzymes of gluconeogenesis. Our analysis showed that the gene expression alterations between mutant and wild type *TP53* patients showed higher expression of glucose transporters that contributes to the higher uptake of glucose, and higher expression of key glycolysis enzymes and pentose phosphate pathway (PPP) enzymes.

We performed the analysis for patients with all mutations and patients with a coding mutation only separately. Almost all patients had a coding mutation in TP53 – only five patient had a silent alteration. This resulted in almost identical effect of a TP53 alteration in all and in coding patients only. The extremely low prevalence of non-coding mutations further emphasize the importance of TP53.

Song and colleagues [30] published a proteomic comparison between normal and breast cancer tissues. Differentially expressed proteins in tumor tissues were related to glycolysis/gluconeogenesis among other pathways. Overexpressed proteins included GAPDH, ENO1, lactate dehydrogenase (LDH), pyruvate kinase (PKM2) and aldolase A (ALDOA). The fact that these proteins were up-regulated in the invasive carcinoma group supports the general concept that rapid growing tumor cells show higher rate of glucose metabolism than normal cells [30]. At the same time mutant *TP53* negatively affected the expression of gluconeogenesis, the glucose synthetic pathway, and the fatty acid oxidation pathway that contributes to the degradation of fatty acids. Recent studies suggest that some of the glucose metabolism enzymes – including GPI and PGK, genes identified in our analysis – are multi-functional proteins. The encoded protein of GPI is also referred to as autocrine motility factor based on an additional function as a tumor-secreted cytokine and angiogenic factor [31]. The glycolysis enzyme PGK participates in angiogenesis by functioning to reduce disulfide bonds in the serine protease, plasmin by tumor cells [32].

Li and colleagues [33] exposed that MDAMB-231 and another mutTP53 cell line (T47D) consumed significantly more glucose and secreted more lactate than the non-tumorgenic and wtTP53 cell line, MCF10A. In the two mutTP53 cell lines (MDA-MB-231 and T47D) 80-94% of the glucose consumed was used to make lactate, while in the MCF10A only the 50% of glucose consumed was used to make lactate. In our study we show that wtTP53 MCF-7 cells display the lowest glycolytic activity in the presence of glucose in amino acid rich media. In DMEM based medium, MCF-7 cells were the most oxidative ones among the investigated cells. JIMT-1 cells exhibited the highest rate of glycolysis and the most pronounced inhibition of oxidative metabolism in the presence of glucose. MDA-MB-231 cells showed a moderate glycolytic activity and mitochondrial activity among these cell lines. None of the cell lines investigated here presented with a high glutamine dependence of oxidation.

During the past years significant efforts have been dedicated to the identification of mediators that selectively eliminate cancer cells based on their metabolic alterations, some of them now close to clinical evaluation [34]. Multiple attempts have been executed to inhibit the rate limiting enzymes of glycolysis, first the unselective inhibitor of hexokinase 2-deoxy-p-glucose (2-DG) was added to patients with glioma [35]. Promising preclinical results showed with other HK inhibitors, 3-bromopyruvate (3-BP) [36] and methyl jasmonate [37] with anticancer effects in mouse models. However, clinical development of 2-DP and 3-BP hexokinase inhibitors has been discontinued – most probably the similarities between malignant cell and normal cell (manly highly proliferative cells) metabolisms will make it difficult to select the appropriate patient cohort eligible for such treatments [38].

Lonidamine, a derivative of indazole-3-carboxylic acid delivered impressive results to inhibit aerobic glycolysis in cancer cells by inhibit HK in clinical trials [39]. Preclinical data showed that inhibition of glucose transporters (GLUT1 inhibition with WZB117 or with silybin) also have antineoplastic effects [40, 41]. Further studies have shown that koningic acid, a potent and selective inhibitor of GAPDH, kills a broad range of highly glycolytic cell lines [42]. Imatinib treatment decreased the activity of both HK and GAPDH in leukemia and myeloid tumors [43]. Pyruvate dehydrogenase kinase (PDK) is often overexpressed in malignant cells, the dichloroacetate (DCA) that originally used in lactic acidosis therapy inhibits PDK, and results mitochondrial defects in cancer cells used by patients with glioblasoma [44]. Oxythiamine is a thiamine antagonist, inhibits both PDK and transketolase (TKT), and responsible for significant anticancer activity [45].

We have to mention a limitation of present study: the investigated cell lines also harbor additional difference in addition to mutations in TP53. At the same time, the primary analysis of the study is based on evaluating a large patient cohorts. The identified genes as well as the study messages holds therefore even without the cell culture experiments. Future studies with additional cell lines will be needed to further validate the effect of each gene on the metabolic profile in TP53 mutant and wild type cell lines.

In summary, here we show in a large patient cohort that *TP53* mutation status simultaneously affects multiple metabolic pathways. The utilization of metabolic inhibitors in cell culture experiments lead to higher glycolytic and mitochondrial activity in *TP53* mutant breast cancer cell lines.

## MATERIALS AND METHODS

### *In silico* analyses

### Database setup

Breast cancer samples from the TCGA database with genome-wide sequencing, gene expression and clinical data for 762 patients were used in the statistical analysis. We analyzed n=547 *TP53* wild type and n=215 *TP53* mutant BC patient samples. Clinical characteristics for all these patients are summarized in Supplementary Table 1.

### NGS data download

Genome-wide sequencing data and RNA-seq data for breast cancer patients were obtained from The Cancer Genome Atlas (TCGA) of the National Cancer Institute (http://cancergenome.nih.gov/) [46]. The aligned TCGA datasets were downloaded from the CGHub repository (website: https://cghub.ucsc.edu/) using the CGHub download client software GeneTorrent (version 3.8.5).

### Mutation calling

Mutation calling and annotation was done with *MuTect* using default parameters. The human reference genomes GRCh37, GRCh37-lite and HG19used for mutation calling were downloaded according to the CGHub website's “Reference Assemblies” guideline (available at https://gdc.cancer.gov/download-gdc-reference-files). To reduce the total number of mutations, we only accepted somatic mutations that were labeled with ‘KEEP’ according to the *MutTect* judgment algorithm, and were present in at least four reads with a minimum of 20 fold read coverage. The identified mutations were annotated with *MuTect* using the most recent versions of the dbSNP (build 139) and COSMIC (version 68) databases. The identified sequence variations were functionally annotated using SNPeff v3.5 [47]. The reference databases used with SNPEff were downloaded with the SNPeff downloader. Mutations were defined as coding mutations in case they changed the amino acid sequence. Coding mutations categories include missense, start/stop loss, start/stop gain, and splice site mutations. Non coding mutations were mutations that do not fall in any of the coding mutation categories.

### Processing of RNA-seq data

RNA-seq data for breast cancer patients was obtained from the TCGA repository for those patients who also had mutation data as well. We downloaded pre-processed level 3 data generated using the Illumina HiSeq 2000 RNA Sequencing Version 2 platform. Expression levels for these samples were determined using a combination of MapSplice and RSEM. Individual patient files were merged in R using into a single database.

### Statistical analysis

Separate ROC analysis was performed for the expression of each gene grouped by TP53 mutation status. The analysis was performed for all mutations, for coding mutations and for silent mutations separately. ROC analysis was completed in the R statistical environment (http://www.r-project.org) using the ROC Bioconductor library (http://www.bioconductor.org) as described previously [48]. Statistical significance was set at FDR adjusted p value<0.05.

### *In vitro* experiments

### Cell lines

Human breast cancer cell lines MCF7, MDA-MB-231 and JIMT-1 were obtained from the American Type Culture Collection (Manassas, VA, USA). Cell lines were cultured in RPMI medium supplemented with 10% fetal bovine serum with 1% penicillin-streptomycin and 0,1% amphotericin (Life Technologies) as described previously [49]. Cells were grown at 37°C under a humidified atmosphere of 95% air and 5% CO_2_.

### STR analysis

Cell line verification was completed by Short tandem repeat (STR) analysis on 9 exact loci of the human genome. Genomic DNA was extracted by DNeasy Kit (Qiagen) according to the protocol provided by the manufacturer. DNA concentration and clarity was confirmed by Nanodrop ND-1000. The SRT analysis was accomplished by Fragment Analysis Facility at Johns Hopkins University (Baltimore, USA). Then, results were compared to the STR database of the German Collection of Microorganisms and Cell Cultures at the Leibniz Institute (http://www.dsmz.de). All the tested cell lines were contamination free.

### RNA isolation

RNA was isolated from 1 × 10^7^ cells in logarithmic growth phase using the Qiagen RNeasy Mini Kit following the manufacturer's protocol (Qiagen GmbH, Hilden, Germany). Isolated total RNA concentrations were measured by NanoDrop ND-1000 spectrophotometer (BCM, Houston, TX, USA). RNA samples were stored at −80°C.

### RT-PCR

RT-PCR assay was performed with Piko™ Thermal Cycler (Thermo Fisher Scientific Inc.) using a OneStep RT–PCR Kit (Qiagen GmbH) as described in the manufacturer's instructions. Gene-specific PCR primers (IDT&Bio-Science Kft., Hungary) were designed by Primer3 software (http://frodo.wi.mit.edu/) and their binding site was verified by NCBI BLAST (http://blast.ncbi.nlm.nih.gov/Blast.cgi). Optimal Tm of each primer was set at 59°C. Amplification cycles were optimized for each gene according to different gene expressions. Then, RT-PCR reaction for wild type and mutant TP 53 cell lines was carried out for each gene with an identical amplification cycle using 0.5 μg of total RNA. Amplification products were separated on a 2% agarose gel stained with GR Safe Nucleic Acid Gel Stain (Excellgen). Results were visualized and recorded by using the Glite900 BW Gel Scanner (Pacific Image Electronics). The analysis was repeated three times. PCR primers, product size and amplification cycles are shown in Supplementary Table 2.

### TP53 mutation validation

Direct DNA sequencing was performed using genomic DNA amplified by polymerase chain reaction. In MDA-MB-231 and JIMT-1 mutation detection was performed in exons 7-8 of *TP53*, while in the case of MCF7 each of the 11 exons were sequenced in full length (Supplementary Figure 1B). Primers were designed to be located in intronic sequences to allow amplification of genomic DNA only. The used primers and primer features are listed in Supplementary Table 3 and illustrated in Supplementary Figure 1A.

The PCR reaction was performed in 25 μl final volume, containing 500 ng of genomic DNA, 10mM of each dNTP (Invitrogen), 10 μM of each of the eight primers, 5 units of Taq polymerase (Invitrogen), 2.5 μl 10x buffer, completing to the final volume with nuclease free H_2_O. The amplification reaction was carried out in thermocycler (Swift Maxi, ESCO) with an initial denaturation step of 3 min at 94°C, followed by 35 cycles consisting of three steps: 94°C for 30 sec, 53°C for 30 sec, and 72°C for 2 min. Annealing temperature was optimized to the primers melting temperature. The last cycle was followed by an extension step of 6 min at 72°C. The PCR product was purified, and DNA sequencing was performed at the Department of Genomic Medicine and Rare Disorders (Semmelweis University, Budapest, Hungary). The DNA sequence was analyzed by BioEdit and Genedoc programs (Supplementary Figure 1A).

### Measurement of cellular respiration and glycolytic activity

Mitochondrial and glycolytic functions of the wtTP53 MCF-7 and the mutTP53 MDA-MB-231 and JIMT-1 cells were monitored using Seahorse XF96 extracellular flux analyzer. (Seahorse Bioscience, North Billerica, MA, USA) Oxygen consumption rate (OCR), as a representation of cellular respiration, and extracellular acidification rate (ECAR), as a measure of glycolytic activity, were assessed in parallel in an assay based on microfluorimetric detection using an XF96 Analyzer, as previously described [50]. Breast cancer cells were plated one day before measurement on Seahorse XF96 cell culture microplates at a 2×104 cells/well density. The growth medium beyond cells was replaced by either the DMEM-based or the EC assay medium 2.5 h before measurement. OCR and ECAR were assessed by the XF96 Analyzer based on determination of O_2_ concentration and pH.

During the measurement, 20 to 26 μl of substrates or inhibitors (at all times prepared freshly in the respective assay medium) were administered into each well to reach the desired final concentrations. Laboratory chemicals were obtained from Sigma (St Louis, MO, USA). L-Glutamine was from Merck (Merck KGaA, Darmstadt, Germany). All additive stock solutions were each time freshly prepared. The composition of the Extracellular assay medium (EC) was: 143 mM NaCl, 3 mM KCl, 1 mM MgCl2, 2 mM CaCl2, 8.3 mM HEPES (pH 7.4). The DMEM-based glucose-free medium was purchased from Seahorse Bioscience (# 102365, North Billerica, MA, USA).

Through OCR and ECAR measurements the effects of adding D-glucose/L-glutamine, oligomycin, 2,4-dinitrophenol (DNP), and antimycin A + rotenone were determined. The use of these metabolic modulators permits determination of several parameters of mitochondrial and glycolytic functions. Key parameters of mitochondrial function include basal respiration, ATP turnover, proton leak and maximal respiration. The difference between the maximal and the basal respiration creates a reserve respiratory capacity of a cell to create ATP via oxidative phosphorylation in response to increased demand of energy (for summary see Figure 3B). Key parameters of cellular glycolysis include glycolysis, and glycolytic capacity (Figure 3D). Glycolytic reserve is the difference between glycolytic capacity and glycolysis that reflects to the cellular capability to rise the glycolytic rate upon increased energy demand.

## SUPPLEMENTARY MATERIAL FIGURES AND TABLES



## References

[1] Warburg O (1956). On respiratory impairment in cancer cells. Science.

[2] Bauer DE, Harris MH, Plas DR, Lum JJ, Hammerman PS, Rathmell JC, Riley JL, Thompson CB (2004). Cytokine stimulation of aerobic glycolysis in hematopoietic cells exceeds proliferative demand. FASEB journal.

[3] Yang X, Borg LA, Eriksson UJ (1997). Altered metabolism and superoxide generation in neural tissue of rat embryos exposed to high glucose. Am J Physiol.

[4] Xu RH, Pelicano H, Zhou Y, Carew JS, Feng L, Bhalla KN, Keating MJ, Huang P (2005). Inhibition of glycolysis in cancer cells: a novel strategy to overcome drug resistance associated with mitochondrial respiratory defect and hypoxia. Cancer research.

[5] Zu XL, Guppy M (2004). Cancer metabolism: facts, fantasy, and fiction. Biochem Biophys Res Commun.

[6] Hsu PP, Sabatini DM (2008). Cancer cell metabolism: Warburg and beyond. Cell.

[7] Hanahan D, Weinberg RA (2011). Hallmarks of cancer: the next generation. Cell.

[8] Vousden KH, Ryan KM (2009). p53 and metabolism. Nat Rev Cancer.

[9] Muller PA, Vousden KH (2013). p53 mutations in cancer. Nat Cell Biol.

[10] Gyorffy B, Bottai G, Lehmann-Che J, Keri G, Orfi L, Iwamoto T, Desmedt C, Bianchini G, Turner NC, de The H, Andre F, Sotiriou C, Hortobagyi GN, Di Leo A, Pusztai L, Santarpia L (2014). TP53 mutation-correlated genes predict the risk of tumor relapse and identify MPS1 as a potential therapeutic kinase in TP53-mutated breast cancers. Mol Oncol.

[11] Mihaly Z, Kormos M, Lanczky A, Dank M, Budczies J, Szasz MA, Gyorffy B (2013). A meta-analysis of gene expression-based biomarkers predicting outcome after tamoxifen treatment in breast cancer. Breast Cancer Res Treat.

[12] Olivier M, Petitjean A, Marcel V, Petre A, Mounawar M, Plymoth A, de Fromentel CC, Hainaut P (2009). Recent advances in p53 research: an interdisciplinary perspective. Cancer gene therapy.

[13] Liu J, Zhang C, Hu W, Feng Z (2015). Tumor suppressor p53 and its mutants in cancer metabolism. Cancer letters.

[14] Ruiz-Lozano P, Hixon ML, Wagner MW, Flores AI, Ikawa S, Baldwin A, Chien KR, Gualberto A (1999). p53 is a transcriptional activator of the muscle-specific phosphoglycerate mutase gene and contributes *in vivo* to the control of its cardiac expression. Cell growth and differentiation.

[15] Bensaad K, Tsuruta A, Selak MA, Vidal MNC, Nakano K, Bartrons R, Gottlieb E, Vousden KH (2006). TIGAR, a p53-inducible regulator of glycolysis and apoptosis. Cell.

[16] Matoba S, Kang J-G, Patino WD, Wragg A, Boehm M, Gavrilova O, Hurley PJ, Bunz F, Hwang PM (2006). p53 regulates mitochondrial respiration. Science.

[17] Kanehisa M, Goto S (2000). KEGG: kyoto encyclopedia of genes and genomes. Nucleic acids research.

[18] Pilkis SJ, Granner D (1992). Molecular physiology of the regulation of hepatic gluconeogenesis and glycolysis. Annual review of physiology.

[19] Hu W, Zhang C, Wu R, Sun Y, Levine A, Feng Z (2010). Glutaminase 2, a novel p53 target gene regulating energy metabolism and antioxidant function. Proceedings of the National Academy of Sciences.

[20] Maddocks OD, Vousden KH (2011). Metabolic regulation by p53. Journal of molecular medicine.

[21] Gyorffy B, Surowiak P, Kiesslich O, Denkert C, Schafer R, Dietel M, Lage H (2006). Gene expression profiling of 30 cancer cell lines predicts resistance towards 11 anticancer drugs at clinically achieved concentrations. International Journal of Cancer.

[22] Warburg O, Wind F, Negelein E (1927). The metabolism of tumors in the body. The Journal of general physiology.

[23] Schwartzenberg-Bar-Yoseph F, Armoni M, Karnieli E (2004). The tumor suppressor p53 down-regulates glucose transporters GLUT1 and GLUT4 gene expression. Cancer research.

[24] Mathupala SP, Heese C, Pedersen PL (1997). Glucose Catabolism in Cancer Cells THE TYPE II HEXOKINASE PROMOTER CONTAINS FUNCTIONALLY ACTIVE RESPONSE ELEMENTS FOR THE TUMOR SUPPRESSOR p53. Journal of Biological Chemistry.

[25] Mathupala SP, Ko YH, Pedersen PL (2009). Hexokinase-2 bound to mitochondria: cancer's stygian link to the “Warburg Effect” and a pivotal target for effective therapy. Seminars in cancer biology.

[26] Šmerc A, Sodja E, Legiša M (2011). Posttranslational modification of 6-phosphofructo-1-kinase as an important feature of cancer metabolism. PloS one.

[27] Sermeus A, Michiels C (2011). Reciprocal influence of the p53 and the hypoxic pathways. Cell Death Dis.

[28] Ide T, Chu K, Aaronson SA, Lee SW (2010). GAMT joins the p53 network: branching into metabolism. Cell Cycle.

[29] Zhang P, Tu B, Wang H, Cao Z, Tang M, Zhang C, Gu B, Li Z, Wang L, Yang Y, Zhao Y, Wang H, Luo J (2014). Tumor suppressor p53 cooperates with SIRT6 to regulate gluconeogenesis by promoting FoxO1 nuclear exclusion. Proc Natl Acad Sci U S A.

[30] Song MN, Moon PG, Lee JE, Na M, Kang W, Chae YS, Park JY, Park H, Baek MC (2012). Proteomic analysis of breast cancer tissues to identify biomarker candidates by gel-assisted digestion and label-free quantification methods using LC-MS/MS. Arch Pharm Res.

[31] Watanabe H, Takehana K, Date M, Shinozaki T, Raz A (1996). Tumor cell autocrine motility factor is the neuroleukin/phosphohexose isomerase polypeptide. Cancer Research.

[32] Lay AJ, Jiang X-M, Kisker O, Flynn E, Underwood A, Condron R, Hogg PJ (2000). Phosphoglycerate kinase acts in tumour angiogenesis as a disulphide reductase. nature.

[33] Li Y, Wang H, Oosterwijk E, Tu C, Shiverick KT, Silverman DN, Frost SC (2009). Expression and activity of carbonic anhydrase IX is associated with metabolic dysfunction in MDA-MB-231 breast cancer cells. Cancer Invest.

[34] Vander Heiden MG (2011). Targeting cancer metabolism: a therapeutic window opens. Nat Rev Drug Discov.

[35] Wolf A, Agnihotri S, Micallef J, Mukherjee J, Sabha N, Cairns R, Hawkins C, Guha A (2011). Hexokinase 2 is a key mediator of aerobic glycolysis and promotes tumor growth in human glioblastoma multiforme. J Exp Med.

[36] Jae HJ, Chung JW, Park HS, Lee MJ, Lee KC, Kim HC, Yoon JH, Chung H, Park JH (2009). The antitumor effect and hepatotoxicity of a hexokinase II inhibitor 3-bromopyruvate: *in vivo* investigation of intraarterial administration in a rabbit VX2 hepatoma model. Korean J Radiol.

[37] Klippel S, Jakubikova J, Delmore J, Ooi M, McMillin D, Kastritis E, Laubach J, Richardson PG, Anderson KC, Mitsiades CS (2012). Methyljasmonate displays *in vitro* and *in vivo* activity against multiple myeloma cells. Br J Haematol.

[38] Galluzzi L, Kepp O, Vander Heiden MG, Kroemer G (2013). Metabolic targets for cancer therapy. Nat Rev Drug Discov.

[39] Di Cosimo S, Ferretti G, Papaldo P, Carlini P, Fabi A, Cognetti F (2003). Lonidamine: efficacy and safety in clinical trials for the treatment of solid tumors. Drugs Today (Barc).

[40] Liu Y, Cao Y, Zhang W, Bergmeier S, Qian Y, Akbar H, Colvin R, Ding J, Tong L, Wu S, Hines J, Chen X (2012). A small-molecule inhibitor of glucose transporter 1 downregulates glycolysis, induces cell-cycle arrest, and inhibits cancer cell growth *in vitro* and *in vivo*. Mol Cancer Ther.

[41] Zhan T, Digel M, Kuch EM, Stremmel W, Fullekrug J (2011). Silybin and dehydrosilybin decrease glucose uptake by inhibiting GLUT proteins. J Cell Biochem.

[42] Colell A, Green DR, Ricci J-E (2009). Novel roles for GAPDH in cell death and carcinogenesis. Cell Death & Differentiation.

[43] Gottschalk S, Anderson N, Hainz C, Eckhardt SG, Serkova NJ (2004). Imatinib (STI571)-mediated changes in glucose metabolism in human leukemia BCR-ABL-positive cells. Clinical Cancer Research.

[44] Michelakis E, Webster L, Mackey J (2008). Dichloroacetate (DCA) as a potential metabolic-targeting therapy for cancer. British journal of cancer.

[45] Comín-Anduix B, Boren J, Martinez S, Moro C, Centelles JJ, Trebukhina R, Petushok N, Lee WNP, Boros LG, Cascante M (2001). The effect of thiamine supplementation on tumour proliferation. European Journal of Biochemistry.

[46] Cancer Genome Atlas N (2012). Comprehensive molecular portraits of human breast tumours. Nature.

[47] Cingolani P, Platts A, Wang le L, Coon M, Nguyen T, Wang L, Land SJ, Lu X, Ruden DM (2012). A program for annotating and predicting the effects of single nucleotide polymorphisms, SnpEff: SNPs in the genome of Drosophila melanogaster strain w1118; iso-2; iso-3. Fly.

[48] Penzvalto Z, Lanczky A, Lenart J, Meggyeshazi N, Krenacs T, Szoboszlai N, Denkert C, Pete I, Gyorffy B (2014). MEK1 is associated with carboplatin resistance and is a prognostic biomarker in epithelial ovarian cancer. BMC Cancer.

[49] Tegze B, Szallasi Z, Haltrich I, Penzvalto Z, Toth Z, Liko I, Gyorffy B (2012). Parallel evolution under chemotherapy pressure in 29 breast cancer cell lines results in dissimilar mechanisms of resistance. PLoS One.

[50] Gerencser AA, Neilson A, Choi SW, Edman U, Yadava N, Oh RJ, Ferrick DA, Nicholls DG, Brand MD (2009). Quantitative microplate-based respirometry with correction for oxygen diffusion. Anal Chem.

