# Supplementary material for: TP53 mutation hits energy metabolism and increases glycolysis in breast cancer
Source: Oncotarget. 2016 Aug 25;7(41):67183–95. doi: 10.18632/oncotarget.11594 (PMC5341867; doi:10.18632/oncotarget.11594)
Supplement: Supplementary file 1 [file oncotarget-07-67183-s001.pdf]

TP53 mutation hits energy metabolism and increases glycolysis in breast cancer

SUPPLEMENTARY FIGURES AND TABLES

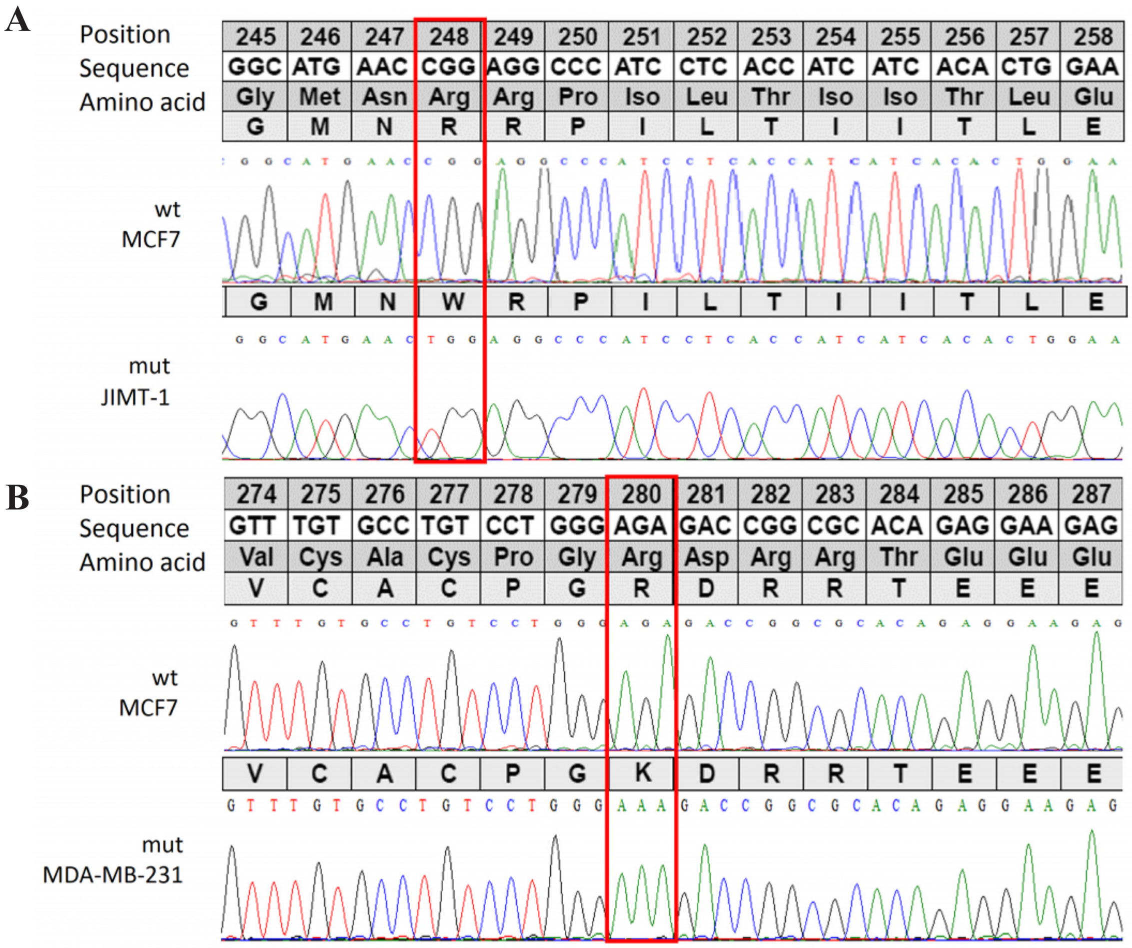

Supplementary Figure S1: Mutations in the *TP53* gene. A. Locations of primers and mutations in the gene structure. Grey boxes represents exons (E1-11), double grey lines between exons represent introns (I1-10). Numbers show the nucleotide length. Vertical arrows show positions of mutations in the two mutant cell lines (JIMT-1 and MDA-MB-231). Horizontal arrows show the primers used in the PCR amplification designed for product sequencing. **B.** Result of the validation of *TP53* mutation in the mutant cell lines by comparison of sequence chromatograms to the wild type *TP53* cell line MCF7.

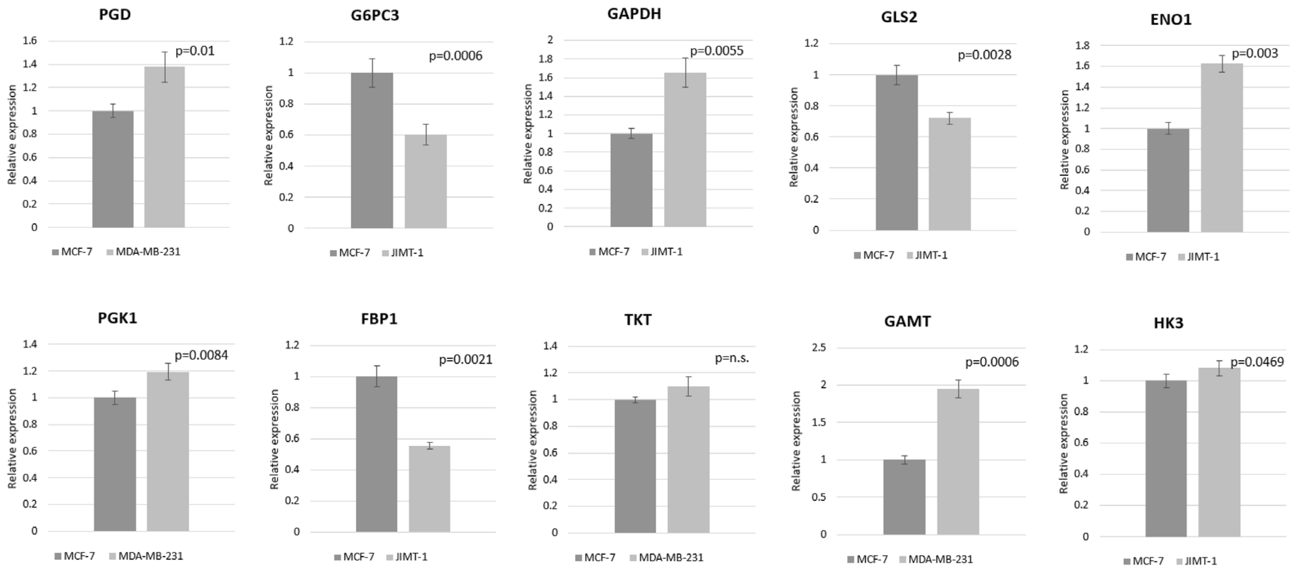

Supplementary Figure S2: Gene expression for a selected set of genes in the cell lines.

Supplementary Table S1: Aggregate clinical characteristics of patient samples included in the study

TP53 MUTATIONAL STATUS	Wild type	Mutated
n	**547**	**215**
**STAGE**		
Stage I	95 (18.0%)	33 (16.2%)
Stage II	303 (57.4%)	115 (56.4%)
Stage III	111 (21.0%)	48 (23.5%)
Stage IV	9 (1.7%)	4 (1.9%)
**T/N/M CLASSIFICATION**		
T1	145 (27.4%)	47 (23.0%)
T2	302 (57.2%)	126 (61.8%)
T3	59 (11.2%)	22 (10.8%)
T4	20 (3.8%)	8 (3.9%)
N0	236 (44.7%)	92 (45.1%)
N1	195 (36.9%)	68 (33.3%)
N2	54 (10.2%)	25 (12.3%)
N3	32 (6.1%)	16 (7.8%)
M0	457 (86.5%)	179 (87.7)
M1	11 (2.1%)	4 (2%)
**MOLECULAR SUBTYPE**		
ER+	433 (82.1%)	96 (47.1%)
ER-	63 (11.9%)	99 (48.5%)
HER2+	93 (17.6%)	50 (24.5%)
HER2-	435 (82.4%)	154 (75.5%)
PGR+	377 (71.4%)	85 (41.5%)
PGR-	117 (22.2%)	111 (54.4%)
Basal	50 (9.5%)	89 (43.7%)
Luminal A	58 (10.9%)	4 (1.9%)
Luminal B	398 (75.4%)	85 (41.7%)
HER2+ER-	22 (4.2%)	26 (12.7%)
**RELAPSE**		
RFS event	21 (3.9%)	10 (4.9%)
Death	51 (9.6%)	22 (10.8%)

Clinical data for some parameters do not add up to 100% because not all data was available for each patient.

Supplementary Table S2: Primer sequences used in the RT-PCR analysis

Gene	No. of transcript variants	Primer – forward	Primer - reverse	Product size (bp)	No. of cycles
HK3	1	gcttcggatgttgagcttgt	gcagccagttgatcatggtt	365	45
GAPDH	4	tcaccagggctgcttttaac	atctcgctcctggaagatgg	191	36
PGK1	1	accttgcctgttgactttgtc	gtgctcacatggctgacttt	341	37
ENO1	2	taccttcatcgctgacctgg	ggccaattacacgactgcaa	392	36
G6PC3	2	gtgggtccatgagtctggtt	gctcctgtgatcatgcagtg	114	37
FBP1	2	aagtcatcctttgccacgtg	aatggttccaacggacacaag	150	38
GLS2	4	cagctcttcgaaggtttgcc	agatggttgaactgcacagc	199	42
GAMT	2	gcagacacacaaggtcatcc	aggttgcagtaggtgaggac	198	42
PGD	3	ggctttgtggtctgtgcttt	aaatcatccacagcttgccc	176	40
TKT	3	caccatggagagctaccaca	tagaggatgggagctgcatg	252	43

Supplementary Table S3: Primers for mutation detection in the genomic *TP53* gene

PRIMER	EXON	INTRON	SEQUENCE	PRODUCT
**TP53-E2-4F**	2-4	2, 3	5'-TCCTCTTGCAGCAGCCAGACTGC-3'	
**TP53-E2-4R**	2-4	2, 3	5'- GGCATTGAAGTCTCATGGAAGC -3'	620bp
**TP53-E5-6F**	5-6	5	5'-CCTGACTTTCAACTCTGTCTCC -3'	
**TP53-E5-6R**	5-6	5	5'-CCAGAGACCCCAGTTGCAAACCAG -3'	400bp
**TP53-E7-9F**	7-9	7, 8	5'-CTCATCTTGGGCCTGTGTTATCTCC -3'	
**TP53-E7-9R**	7-9	7, 8	5'-CCACTTGATAAGAGGTCCCAAGACTTAG-3'	760bp
**TP53-E10-11F**	10-11	10	5'-CCTCCTCTGTTGCTGCAGATCC-3'	
**TP53-E10-11R**	10-11	10	5'-GGCTGTCAGTGGGGAACAAGAAG-3'	1110bp
